# Potential Probiotic Strains of *Saccharomyces* and Non-*Saccharomyces*: Functional and Biotechnological Characteristics

**DOI:** 10.3390/jof7030177

**Published:** 2021-03-02

**Authors:** Pilar Fernández-Pacheco, Cristina Pintado, Ana Briones Pérez, María Arévalo-Villena

**Affiliations:** 1 Analytical Chemistry and Food Technology Department, Faculty of Chemical Sciences and Technologies, Castilla-La Mancha University, 13071 Ciudad Real, Spain; Ana.Briones@uclm.es (A.B.P.); Maria.Arevalo@uclm.es (M.A.-V.); 2 Inorganic Chemistry, Organic Chemistry and Biochemistry Department, Castilla-La Mancha University, 45071 Toledo, Spain; Cristina.Pintado@uclm.es

**Keywords:** *Saccharomyces* and non-*Saccharomyces* probiotic yeasts, functional characteristics, biotechnological potential, Caco-2/TC7 cells, prebiotic metabolisms, assimilation of cholesterol, enzymatic activity, antioxidant activity, antifungal resistance, attenuation

## Abstract

Due to the evident demand for probiotic microorganisms, a growing number of scientific studies have involved the preliminary selection of new strains, but deeper studies for knowing specific functional and biotechnological properties are needed. In the present work, twenty yeasts (*Saccharomyces* and non-*Saccharomyces*) with potential probiotic characteristics, selected in previous works, were evaluated. The following assays were realized: adhesion to Caco-2/TC7 cells, prebiotic metabolisms, assimilation of cholesterol, enzymatic and antioxidant activity, and antifungal resistance. In addition, the effect of ultrasonic treatment was evaluated for attenuating the cultures before their possible incorporation into a food or supplement. In all of the cases, the unique commercial probiotic yeast (*S. boulardii* CNM I-745) was used as positive control. Results show different capabilities depending on the property studied. In general, no *Saccharomyces* yeasts were better in the adhesion to Caco cells, prebiotic metabolism, and presented higher variability of enzymatic activities. The ones related to cholesterol assimilation and antioxidant capability did not show a marked trend, and with respect to the attenuation process, the *Saccharomyces* yeasts were more resistant. For selecting the potential probiotic yeasts with better balance among all characteristics, a principal component analysis (PCA) was carried out. The most promising yeasts for use as health-promoting probiotics are *Hanseniaspora osmophila* 1056 and 1094, *Lachancea thermotolerans* 1039, and *S. cerevisiae* 3 and 146.

## 1. Introduction

Probiotics are live microorganisms with beneficial properties. Their intake in adequate amounts may confer health benefits, according to the FAO/WHO guidelines of 2002.

Most probiotics are isolated from the gut microbiota of healthy individuals or from dairy foods, which are considered a safe isolation source of microorganisms for product development. The market for biofunctional products is continuously in need of the implementation and diversification of the available products. For this purpose, a growing number of scientific studies has involved the selection of new strains with different and specific functional properties, and new microbial groups are associated with these features. Among them, *Saccharomyces* and non-*Saccharomyces* yeasts became promising probiotic candidates over the last decade. These new microorganisms are usually isolated from novel sources, such as traditional fermented foods, traditional fermented drinks, vegetables, fruit juices, fruits, and grains [1,2,3,4].

Cultures and species of *Saccharomyces* normally do not pose any health risk; thus, are designated as generally recognized as safe (GRAS) organisms [5,6], although some studies show the influence of *Saccharomyces cerevisiae* strains from insects on the immune system [7]. However, this genus is the most common and important microorganism associated with fermented foods throughout history. Moreover, a recent report from Li et al. [8], among others, has confirmed the GRAS status of certain non-*Saccharomyces*.

To evaluate the properties of putative probiotic strains, a preliminary in vitro assessment is recommended [9]. This assessment has traditionally paid special attention to the ecological origin of the strains, their tolerance to the hostile conditions of the gastrointestinal transit, and their ability to adhere to intestinal surfaces [10]. In addition, several criterial stabilities during the manufacturing processes must be taken into account [11]. Nevertheless, a deeper study of the functional and biotechnological properties is necessary for selection of new probiotic microorganisms.

Among their potential benefits, yeasts can be used to eliminate cholesterol by assimilation [3] and prevent cardiovascular diseases, considering the failure of current strategies, such as dieting and pharmacological agents [12].

They also can produce major metabolites, such as vitamins [3] and/or enzymes: esterase, protease, or lipase improve digestion by contributing to the absorption of nutrients. A deficiency of digestive enzymes can cause malnutrition, low rate of body development, allergic conditions, digestive disorders, fatigue, liver hypertrophy, or intolerance to certain foods. Another possible characteristic is the antioxidant capability against reactive oxygen species, such as superoxide (O_2_^−^) and hydroxyl (OH) radicals, H_2_O_2_, and singlet oxygen (^1^O_2_), which damage cellular components by oxidizing lipids, proteins, and nucleic acids [13].

On the other hand, probiotics are tackling developing gastrointestinal symptoms, such as abdominal distension, pain, flatulence, and diarrhea, related to lactase metabolism and indigestible oligosaccharides present in pulses and legumes, such as melibiose, raffinose, and stachyose. These kinds of compounds (prebiotics [14]) promote the growth of the microorganisms present in the intestine, resulting in health benefits, such as enhanced antagonism microbiota against foreign microorganisms and contribution of the recolonization of the mucosa, after treatment with antibiotics, generation of H_2_ and CO_2_, as well as short-chain fatty acids that should inhibit the growth of pathogenic organisms.

Otherwise, and from a more practical point of view, the adhesion of the potential probiotic to epithelial cells must be studied as a previous step to the human assays. Moreover, during the inclusion in a food matrix, moderate sonication can be used to attenuate the microorganisms and consecutively introduce them into the product without risks [15,16,17].

Based on all of the explained—and due to the lack of information related to biotechnological characterization of potential probiotics—in the present work, twenty yeast strains isolated from different food ecosystems (winery, distillery, and fermented vegetables) whose probiotic features had previously been studied [18,19,20], were exhaustively characterized by study of their adhesion to Caco-2/TC7 cells, use of prebiotics and lactose, the assimilation of cholesterol, resistance to antifungals, enzymatic and antioxidant activities, and the effect of ultrasonic treatment (attenuation) on cellular viability and vitality.

## 2. Materials and Methods

### 2.1. Yeast Strains

Ten *Saccharomyces cerevisiae* and ten non-*Saccharomyces* (belonging to the genera *Pichia, Lachancea, Hanseniaspora, Candida,* and *Zygosaccharomyces*) yeast strains isolated from different food ecosystems were used (Table 1).

All of them were selected according to the probiotic potential in vitro shown in other works [19,20,21] for presenting a high resistance to the gastrointestinal tract conditions and for showing the most adequate values of the kinetic parameters. They also presented good adherence characteristics (results of autoaggregation, hydrophobicity, and biofilm formation studies), among them and with the mucosa. In addition, a commercial probiotic yeast (*Saccharomyces boulardii*) was used as a positive control.

For obtaining fresh cultures, each strain was grown for 24 h at 30 °C in YPD (Yeast Extract Peptone Dextrose) broth (1% yeast extract, 2% peptone and 2% dextrose) (Pronadisa-Conda, Madrid Spain). In some experiments, it was necessary to determinate the yeasts population for carry out the inoculation with 10^6^ cfu/mL. It was done using the Thoma chamber (Preciss Europe, Laboroptik Ltd., Lancing,). Once calculated, the adequate volume of broth was harvested by centrifugation at 20 °C (1800× *g* for 5 min). Pellet was washed twice with saline solution (0.9%, Sigma, St. Louis, MO, USA) and suspended in the corresponding medium of each assay.

Biological triplicates were performed in all experiments.

### 2.2. Yeast Adhesion to Caco-2/TC7 Cells

The mechanisms by which probiotics exert positive biological effects in the body are not yet fully understood, but adherence to the intestinal mucosa is often used to explain their mode of action [22].

Adhesion assays were performed following Diosma et al. [23] with slight modifications. Caco-2/TC7 cells, derived from a human epithelial colorectal adenocarcinoma, were routinely grown in Dulbecco’s Modified Eagle Minimal Essential Medium (DMEM, Lonza, BioWhittaker™, Belgium) supplemented with 10% (*v*/*v*) fetal bovine serum (FBS), 100 U/mL penicillin-streptomycin and glutamine (2 mM), following the procedure described by Golowczyc et al. [24]. Cells in subculture passage 23 were seeded at a concentration of 2.5 × 10^5^ cells per well in 24-well tissue culture plates. Overnight cultures of yeasts were resuspended in 0.5 mL of Dulbecco’s Modified Eagle Minimal Essential Medium (DMEM, BioWhittaker™, Belgium) with a concentration of 10^6^ cfu/0.5 mL. Each strain suspension was added onto the monolayer culture of Caco-2/TC7 (differentiated cells).

The plates were incubated for 1 h at 37 °C in a 5% CO_2_, 70.5% rh. The monolayer was then washed three times with PBS and lysed in 0.5 mL of sterile distilled water. To determine the number of viable yeasts associated with the Caco-2/TC7 cells, the appropriate dilutions in saline solution were plated on petrifilm YM (3M Petrifilm^TM^, St. Paul, MN, USA) and after 2 days at room temperature, the colonies were counted. Results were expressed as the percentage of yeasts adhered to the Caco-2/TC7 cells, with respect to the population inoculated.

### 2.3. Prebiotic Metabolisms

Certain compounds, such as fructooligosaccharides, galactooligosaccharides, and xylooligosaccharides, among others, are known to promote the development of bacteria probiotics [14]; therefore, it is of keen interest to know which nutrients are the most suitable for these strains.

#### 2.3.1. Aerobic Conditions

Different prebiotic solutions were prepared with prebiotic (10 g/L), yeast extract (10 g/L), and peptone (20 g/L). The prebiotics used were melibiose, raffinose, xylan, trehalose, pectin, beta-glucan, cellulose, cellobiose, and inulin (Sigma, St. Louis, MO, USA). Two controls were used: the yeast control (yeast with yeast extract and peptone) and medium control (prebiotic, yeast extract, and peptone).

In parallel, and to deplete the residual sugar, the overnight cultures were incubated for 6 h at 30 °C in saline solution (0.9%). Then, they were inoculated, at a concentration of 10^6^ cfu/mL, into 190 μL of prebiotic solution using 24-well plates and incubated at 30 °C.

The assimilation verification was performed through readings on a microplate spectrophotometer (ELx808™ Absorbance Microscope Reader), with measurements at 600 nm for 5 days. It was assumed that a strain had used the prebiotic when there was an absorbance increment of at least 0.55 with respect to the yeast control.

#### 2.3.2. Anaerobic Conditions

The same protocol detailed in Section 2.3.1 was used, but the plates with the prebiotic solutions (once inoculated) were sealed with Vaseline.

### 2.4. Assimilation of Cholesterol

Some of the beneficial effects of probiotics in the clinical field may be their ability to assimilate cholesterol, as an alternative to the prevention of cardiovascular diseases [25].

YPD growth medium was used, supplemented with bile salts (5%, Oxoid, Basingstoke, UK) and cholesterol (0.2%, Sigma-Aldrich Co., St. Louis, MO, USA). Then it was inoculated with 10^6^ cfu/mL of each strain for 48 h at 30 °C. Cholesterol was measured using the process described by Gilliland et al. [26]. To determine the assimilated cholesterol in the medium, the absorbance was read at 600 nm (Jasco V-530 spectrophotometer, Madrid, Spain) at 0 h and 48 h. Percentage assimilation was calculated as follows:

(1) $$\left(1-\left(\frac{A48\;h}{A0\;h}\right)\right)\times 100\%$$

### 2.5. Enzymatic Activity

Another benefit of probiotics could be their contribution to digestion, providing different enzymes that facilitate the degradation of macronutrients, improving the assimilation of nutrients.

The study of certain enzymatic activities (alkaline phosphatase, esterase (C 4), esterase lipase (C 14), lipase, leucine arylamidase, valine arylamidase, cystine arylamidase, trypsin, α-chymotrypsin, acid phosphatase, and naphthol-AS-BI-phosphohydrolase) was carried out with a semi-quantitative standardized API ZYM micro method (BioMérieux, Madrid, Spain).

Every microwell was inoculated with 65 µL of a suspension of 10^6^ cfu/mL. It was incubated for 4 h at 37 °C. After this time, the reagents ZYM A (tri-hydroxy-methyl-amino-methane, HCl, and lauryl sulfate) and ZYM B (fast blue BB and 2-methoxy-ethanol) were added, and the results were taken 15 min later.

In addition, lactase activity was also evaluated, both in aerobic and anaerobic conditions. The methodology used was the one described in Section 2.3.1 and Section 2.3.2 respectively, using lactose as a carbon source (Sigma Chemical, St. Louis, MO, USA).

### 2.6. Antioxidant Activity

Other metabolic functions of probiotics are based on secreting functional bioactive molecules to protect the human body, for example, against free radicals [13].

Two assays were carried out to study the antioxidant activity: free radical scavenging and catalase activity.

Free radical scavenging was assessed through a colorimetric DPPH (1,1-diphenyl-2-picrylhydrazyl) test, according to the method described by Brand-Williams et al. [27], with some modifications.

In short, aliquots of 0.4 mL of yeast cell suspensions (with a population of 10^6^ cfu/mL) were mixed with 1.6 mL of DPPH 6 × 10^−5^M methanolic solution [28]. The mixture was shaken vigorously and then incubated at 30 °C for 30 min in the dark. Absorbance at 517 nm was measured (Jasco V-530 spectrophotometer, Madrid, Spain) at “time 0” (A0) and after incubation (AF). The scavenging free radical percentage was calculated as follows:

(2) $$\left(1-\left(\frac{\mathrm{AF}}{A0}\right)\right)\times 100\%$$

The results were expressed as free radical scavenging percentages.

The second assay, catalase activity, was done by adding 3% hydrogen peroxide (*v*/*v*) to 300 μL of a 10^6^ cfu/mL suspension of each yeast strain, according to the Whittenbury [29] method. The presence of bubbles indicated positive catalase activity.

### 2.7. Antifungal Resistance

Knowing the behavior of yeasts used as probiotics against antifungals opens the possibility of their use during treatments with these drugs.

Resistance to certain common antifungals was evaluated. The methodology used was as follows. Each yeast strain was seeded on YPD agar Petri dishes using the microbial turf technique, and then a drop of 5 µL of each antifungal solution was added. The plates were incubated at 30 °C for 48 h. The sensitivity of the strains against the antifungal compounds was determined by measuring the diameter (cm) of total (complete absence of w growth) and partial (slight growth) inhibition zones.

The antifungals used were nystatin (21 mg/mL) (Mycostatin^®^, Bristol-Myers Squibb, S.A.), ciclopirox olamine (1 mg/mL) (CycloChem^®^, Ferrer), clotrimazole (10 mg/mL) (Canesten^®^, Bayer), and fluconazole (150 mg/mL) (Fluconazol^®^, Cinfa). All of them were purchased in a local pharmacy (Ciudad Real, Spain).

### 2.8. Attenuation

Moderate sonication can be used to attenuate the microorganisms and consecutively introduce them into a food matrix [15].

#### 2.8.1. Setup of Method

The best protocol would be one that makes microorganisms lose their vitality, but not viability. The conditions tested were a combination of the population (10^6^ and 10^7^ cfu/mL), the amplitude (60% and 80%), the time in the sonication process (1, 2, and 3 min), and pulses (absence and presence of pulses, 2 s on and 10 s off) (Q700, Qsonica Sonicators, Newtown, CT, USA). Before each treatment, the sonicator probe was washed with sterile distilled water, and immediately after processing, samples were cooled in ice. Loss of viability was evaluated by seeding on a plate.

#### 2.8.2. Attenuation Effects

One experiment with six yeasts (two *Saccharomyces* strains (3 and 24) and four non-*Saccharomyces* strains (1003, 1019, 1082, and 1090)) was carried out to check whether attenuation affected vitality as well as viability. The effect was measured after sonication and kinetic results were compared with the ones obtained from the culture with the same population without sonication.

Viability was obtained by account in YPD agar and vitality was quantified by means of impedance values (Microtrac 4200, SY-LAB instruments, Neupurkersdorf, Austria). Every 2 min, the MicroTrac measures the variation of impedance of the KOH solution at 0.2%, which takes the generated CO_2_ from the YPD broth inoculated with the strain under study.

Negative and positive controls (samples without yeast and samples with non-sonicated yeasts, respectively) were carried out.

### 2.9. Statistical Analysis

The results were analyzed through different statistical analyses. To determine significant differences between the strains, a one-way analysis of variance (ANOVA) was carried out on every experiment, along with Student’s *t*-test or Duncan test (*p* < 0.05). After all of the experiments, a principal component analysis (PCA) was performed on the correlation matrix and used as a selection method. The software programs used were Excel 2013 (Microsoft Corporation) and SPSS (IBM SPSS Statistics v20).

## 3. Results and Discussion

### 3.1. Yeast Adhesion to Caco-2/TC7 Cells

The adhesive capacity of selected yeasts was examined in vitro with the Caco-2/TC7 intestine-derived cell line. A clear difference between the behavior of *Saccharomyces* and non-*Saccharomyces* strains was observed: the latter had a higher capacity for adhesion.

**Figure 1:**
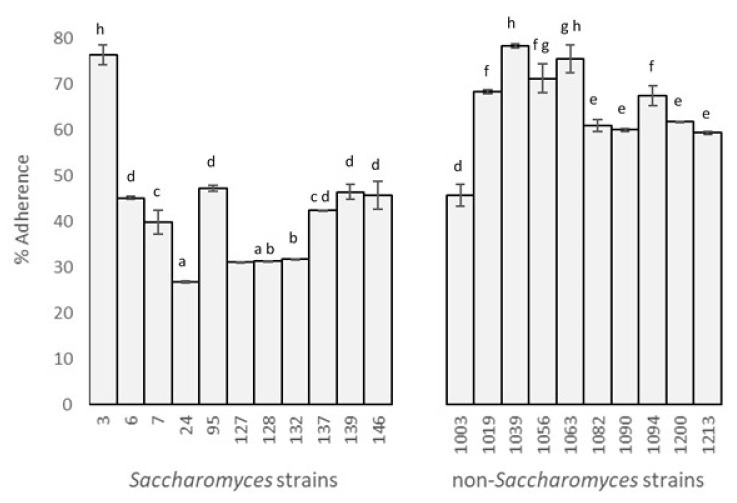
Percentages of yeast adhesion to Caco-2/TC7 cells (different letters indicate significant differences between strains).

Percentages of adhesion (Figure 1) showed values of between 26.78% (*S. boulardii*) and 78.38% (*L. thermotolerans*). According to the Duncan test (α = 0.05, F = 116.39, *p* = 0.00), there were eight significantly different, sorted by decreasing percentage of adhesion. The highest results were for one *Saccharomyces* and two non-*Saccharomyces* strains (3, 1039, and 1063) with values of 76.37 ± 2.10%, 78.30 ± 0.49%, and 75.52 ± 2.96%, respectively. The next three higher groups include the rest of the non-*Saccharomyces*, except for strain 1003 (*P. kudriavzevii*).

All *Saccharomyces*, except for strain 3, were included in the groups with lowest values. Surprisingly, the commercial probiotic, used as a positive control, presented a low percentage of adhesion.

A high correlation has been found between adhesion capacity and biofilm formation after the gastrointestinal tract (a capacity that has been examined for the same yeasts in previous studies [19,20]). This could translate to successful adhesion to the mucosa in humans.

Consistent with these results, Diosma et al. [23] reported 60% adhesion for non-*Saccharomyces* and less than 30% for *Saccharomyces* strains, and Kumura et al. [30] found 80% adhesion for *Kluyveromyces marxianus*. On the other hand, the results presented by Golowczyc et al. [24] for eight *Lactobacillus* sp. strains isolated from kefir grains were worse than the ones presented here.

**Figure 2:**
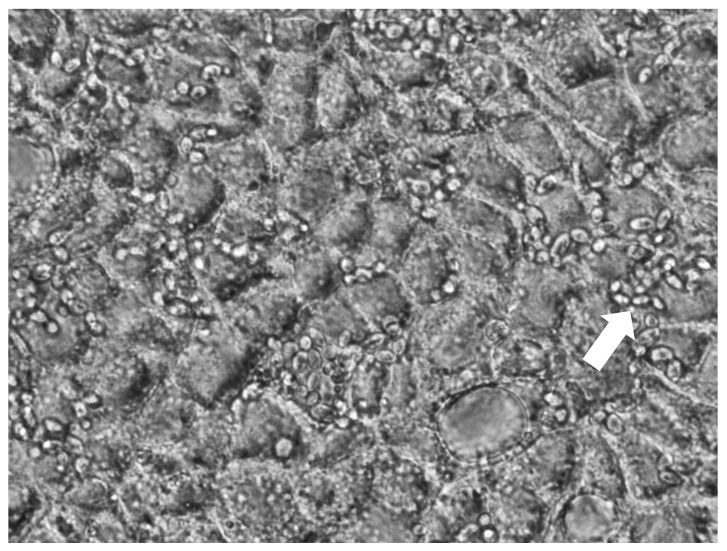
Adhesion of yeast strain 3 to Caco-2/TC7 cells. White arrow points to yeast cells.

Regarding the growth on the epithelial cells, the morphology was unicellular in all cases and no hyphae were observed. This is key for the aspect in the safety of probiotic microorganisms (Figure 2) [9].

### 3.2. Prebiotics Metabolism

#### 3.2.1. Aerobic Conditions

The results of assimilation for the nine prebiotics studied showed that the capability of non-*Saccharomyces* yeasts was higher than that observed in *Saccharomyces*. In most cases, the prebiotics were absorbed within 24 h (Table 2).

The most assimilated prebiotics were raffinose and pectin, followed by cellobiose and trehalose. All strains were able to use the raffinose within 24 h, although according to Kurtzman et al. [31], *Saccharomyces* should be the only one capable of using it. In addition, other prebiotics were assimilated, such as inulin, melibiose, xylan, and cellobiose. The National Collection of Yeast Cultures (NYCN) (Norwich, UK) contains several *S. cerevisiae* strains that also use trehalose and raffinose in aerobic conditions from bakeries, kefir, or distilleries.

Out of the non-*Saccharomyces* strains, only *H. osmophila* (1094) was able to assimilate all prebiotics within 24 h; it achieved a high absorbance increment in the assimilation of cellulose, inulin, melibiose, xylan, trehalose, cellobiose, and beta-glucans (in comparison with the rest of the strains studied). The other *H. osmophila* strain (1056) showed assimilation of only raffinose and beta-glucans, in both cases within 24 h. Two *H. osmophila* strains from NCY showed the ability to assimilate cellobiose obtained from ripe grapes and soil. Excellent results were also shown by some strains of the *Pichia* genus: strains 1003 and 1090 were able to assimilate seven prebiotics in the first 24 h, and strain 1082 and *C. vini* (1063) assimilated six prebiotics within 24 h.

#### 3.2.2. Anaerobic Conditions

In contrast with aerobic conditions, only seven prebiotics were fermented under anaerobic conditions (Table 2). In general, yeasts need longer periods to use prebiotics as a carbon source in anaerobic conditions. The most fermented prebiotic was trehalose and the strain capable of fermenting most compounds was *H. osmophila* (1056), followed by *L. thermotolerans* (1039) and *C. vini* (1063), which fermented three prebiotics within 48 h. Other non-*Saccharomyces* that yielded good results were *P. kudriavzevii*, *H. osmophila,* and *Z. bailii*, which fermented cellobiose, trehalose, raffinose, xylan, and melibiose, although these activities had not been reported by Kurtzman et al. [31].

Unlike under aerobiosis conditions, only two strains of *S. cerevisiae* were able to ferment raffinose while nine of the eleven strains were able to ferment trehalose and five of them the cellobiose, although, according to Kurtzman et al. [31], *S. cerevisiae* only reported to ferment raffinose, as in the NCYC catalogue.

### 3.3. Assimilation of Cholesterol

The highest percentages of assimilation were from five strains of *Saccharomyces* (3, 24, 95, 132, and 146), with the commercial probiotic being one of them, although this ability was not discriminant between the strains (α = 0.05, F = 2.87, *p* = 0.10). All of them were able to remove between 78.52 ± 1.8% and 88.92 ± 2.4% of cholesterol from YPD broth. These assimilation values were similar to the data obtained by Psomas et al. [32] for some *Saccharomyces* sp. strains and higher than those obtained by Chen et al. [33] (between 0% and 45.70 ± 2.6% for non-*Saccharomyces* strains isolated from raw milk).

### 3.4. Enzymatic Activity

In the present study, specific digestive enzyme activities were assessed. The results are shown in Table 3. The majority of the *Saccharomyces* strains presented the same enzymatic profile (esterase C4, esterase C8 lipase, leucine arylamidase, acid phosphatase, and phosphohydrolase). Regarding the non-*Saccharomyces* strains, they presented a higher variability, presenting different activities (esterase (C4), esterase lipase (C8), leucine arylamidase, valine arylamidase, cystine arylamidase, acid phosphatase, and phosphohydrolase). A similar enzymatic profile was found by Psomas [34] in strains of *S. cerevisiae,* and in non-*Saccharomyces* isolates from infant feces and from feta cheese with some probiotic properties, where all strains exhibited acid phosphatase activity, esterase and esterase-lipase activity and none of the strains presented trypsin or α-chymotrypsin activity. In addition, other studies [35] with selected probiotics of lactobacilli derived from chicken feces found similar results for leucine, arylamidase, cystine, arylamidase, acid phosphatase, and naphthol-AS-BI-phosphohydrolase activities. Therefore, *a priori*, these activities give them an adequate profile as probiotic strains. They can enhance enzyme activities since can maintain the gut homeostasis [36].

Regarding lactose activity, none of the strains was able to assimilate or ferment lactose.

### 3.5. Antioxidant Activity

All of the yeasts exhibited antioxidative activity through scavenging free radical from the medium. The values obtained were between 12.08% and 33.71% inhibition, and two significantly different groups were found (α = 0.05, F = 5.62, Significance = 0.00). The second group, which presents the best properties, includes all of the strains except some non-*Saccharomyces* (1019, 1039, 1056, and 1213), which are in the first group. The strains with the highest percentage of reduction of DPPH were two *Saccharomyces* strains (3 and 6) and one *P. kudriavzevii* (1200) with values of 33.71%, 32.66%, and 33.42%, respectively. Trotta et al. [27] and de Lima et al. [37] also found percentages of antioxidant activity in the same range for *S. cerevisiae, S. boulardii,* and *Debaryomyces hansenii*.

In addition, all strains were positive for catalase; that is, these yeasts have an enzyme defense system capable of transforming the reactive oxygen species of hydrogen peroxide into O_2_ and H_2_O [38,39]. This activity could be effective in alleviating inflammation in an inflammatory bowel disease, as reported by Tomusiak-Plebanek et al. [40].

### 3.6. Antifungal Resistance

All of the strains showed a degree of inhibition against the antifungals used, except for 1056 (*H. osmophila*), which, when in contact with ciclopirox olamine, did not show any halo of inhibition, and is therefore totally resistant to this antifungal.

**Figure 3:**
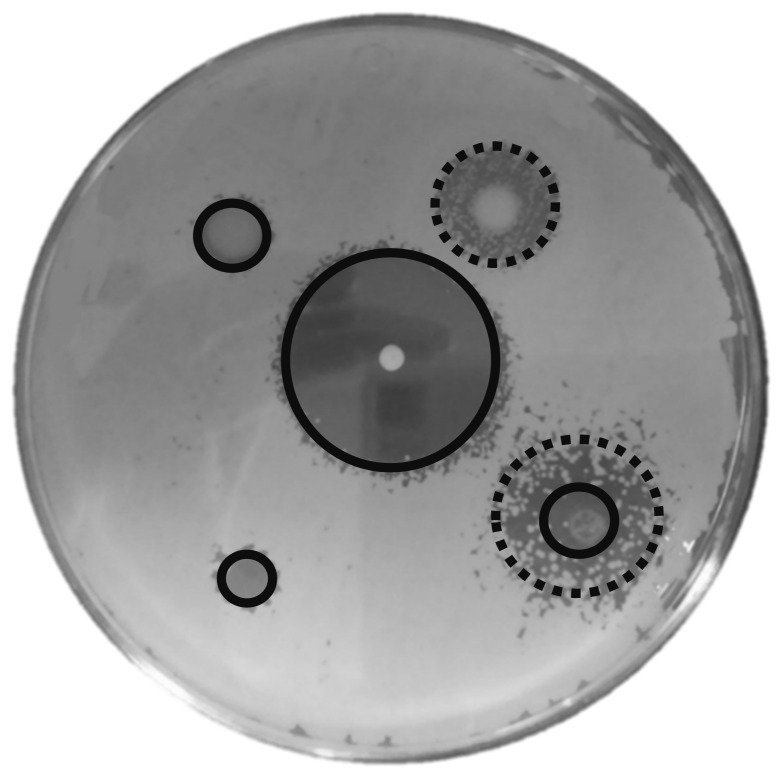
Inhibition halo of yeast growth in presence of antifungals assayed. Black line/circle represents total inhibition of the yeast growth, and dotted line/circle represents partial inhibition of the yeast growth.

**Figure 4:**
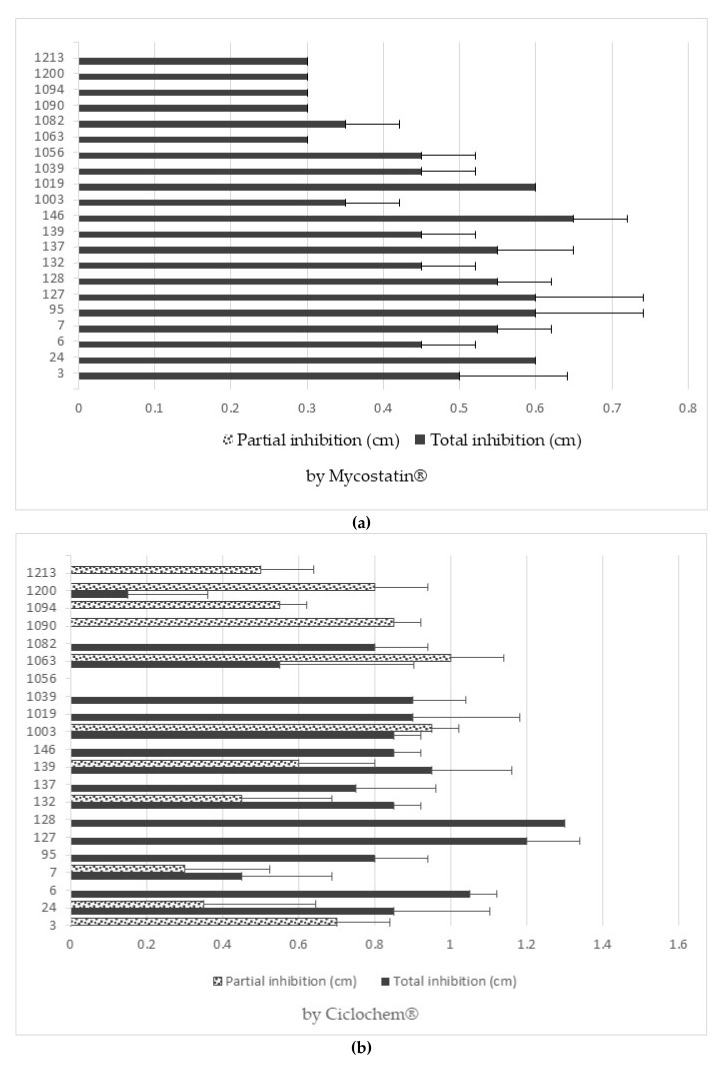

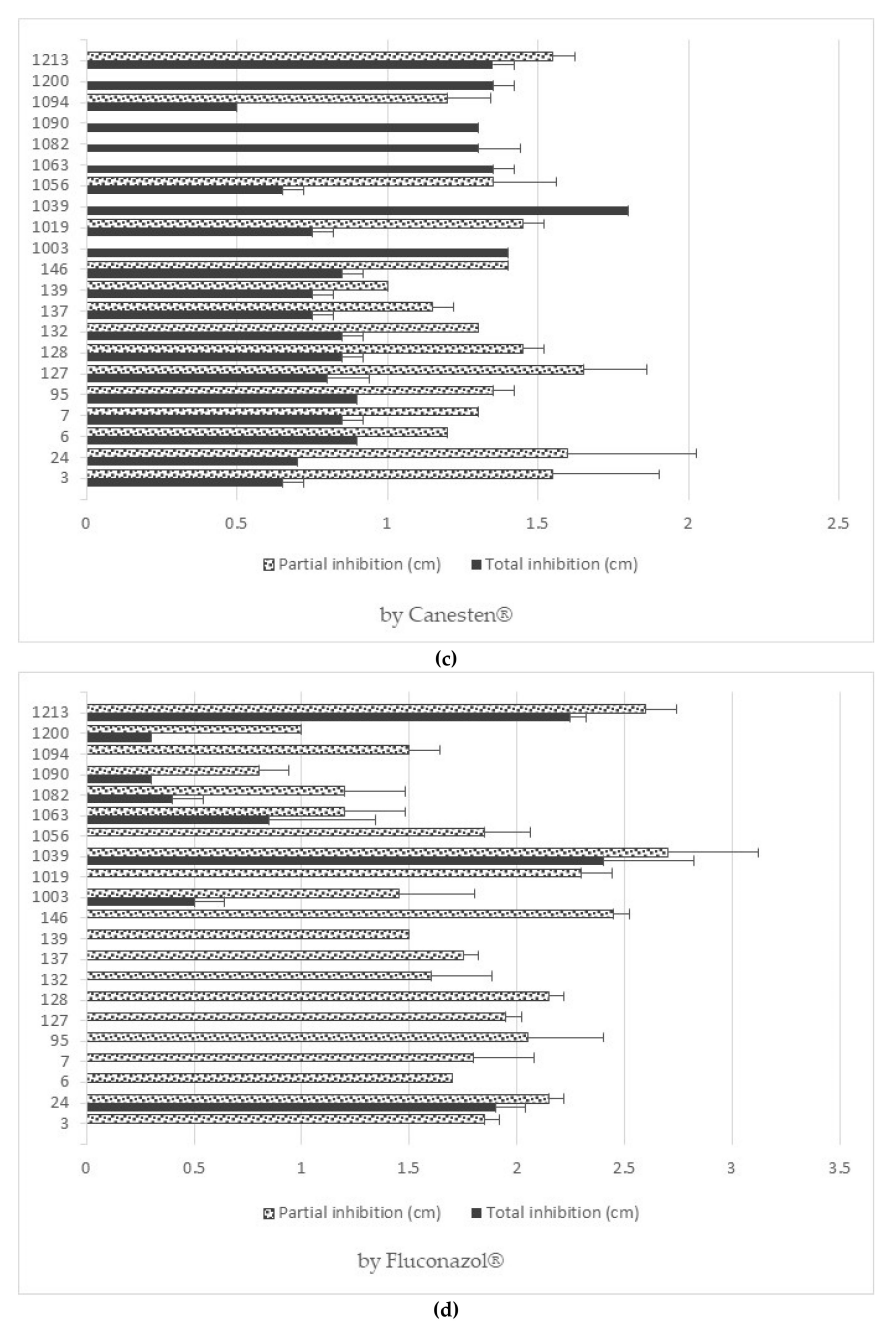
Partial and total inhibition of each yeast against Mycostatin^®^ (**a**), Ciclochem^®^ (**b**), Canesten^®^ (**c**), and Fluconazol^®^ (**d**).

Figure 3 shows clear examples of total and partial inhibition. In the case of nystatin and ciclopirox olamine, the strains with the highest resistance (and therefore the smallest halo of inhibition) were non-*Saccharomyces*. On the other hand, with clotrimazole and fluconazole, *Saccharomyces* strains were the most resistant. All data are shown in Figure 4a,b.

The yeasts studied could continue to exert their beneficial effect on the organism without being affected by the consumption of antibiotics or even certain antifungal drugs (especially strains 3, 1056, and 1094).

### 3.7. Attenuation

#### 3.7.1. Setup of the Method

This was done to find the most suitable parameters, taking into account the compromise between maintaining viability and the loss of vitality. The sonication time was the factor that most affected the yeasts’ viability after the sonication process (Table 4). The attenuation conditions chosen were 60% amplitude and a total time of 2 min (with pulses of 2 s on and 10 s off), since the loss of viability produced was not significant.

#### 3.7.2. Attenuation Effects

The previous experiment, carried out with six strains, indicated that the loss of vitality was not derived from a loss of viability. Student’s *t*-test (α = 0.05) showed that there were significant differences between the vitality of the sonicated samples and that of the non-sonicated samples from the same population.

**Figure 5:**
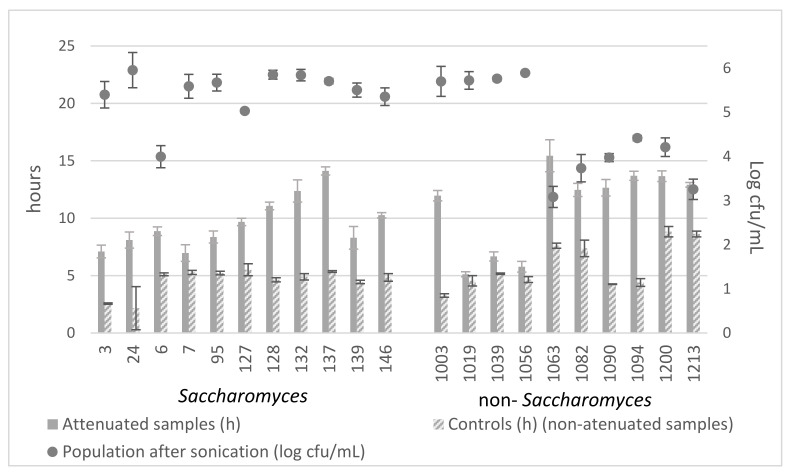
Yeast viability and vitality after the attenuation relative to the corresponding controls.

With the selected sonication conditions (60% amplitude for a total period of 2 min with pulses of 2 s on 10 s off), variable attenuation was achieved among the different strains (Figure 5).

Overall, without subjecting the strains to sonication, the strains of *Saccharomyces* had a similar vitality (they started growing between 2.17 h and 5.51 h), while for the non-*Saccharomyces* strains, there was more variation (between 3.27 h and 8.83 h).

Comparing the final effect of attenuation on each strain (α = 0.05, F = 69.22, Significance = 0.00), it was observed that the strain with the lowest vitality was 1063 (15.44 h) with significant differences, followed by the strains 137 (14.05 h), 1094 (13.70 h), and 1200 (13.67 h), among which there were no significant differences. Even without sonication, strains 1063 and 1200 already showed low vitality at 7.61 h and 8.83 h, respectively.

Comparing the sonicated samples with their corresponding controls (samples not subjected to sonication), a greater attenuation effect was achieved in strains 137, 1003, 1090, and 1094 (8.69, 8.70, 8.40, and 9.30, respectively) without significant differences between them (α = 0.05, F = 63.66, Significance = 0.00).

Regarding viability, the strains of *Saccharomyces* were quite resistant. The loss of viability fluctuated between 0.76% and 16.18% with strain 6 being an outlier, with a loss of 33.43%. Non-*Saccharomyces* yeasts were more susceptible to treatment. There were two groups; the most resistant (1003, 1019, 1039, and 1056) displayed percentages of loss in viability between 1.80% and 5.02%, and the rest displayed percentages of between 26.42% and 49.11%.

Therefore, based on a balance between minimum loss of viability and maximum final attenuation, the best strain is 132, followed by 137, both of them *Saccharomyces*.

### 3.8. Selection of Strains

To select the most promising yeast strains, a PCA analysis was carried out for both *Saccharomyces* and non-*Saccharomyces* strains. For the final decision, the results of the adhesion to Caco-2/TC7 cells test were also taken into account.

**Figure 6:**
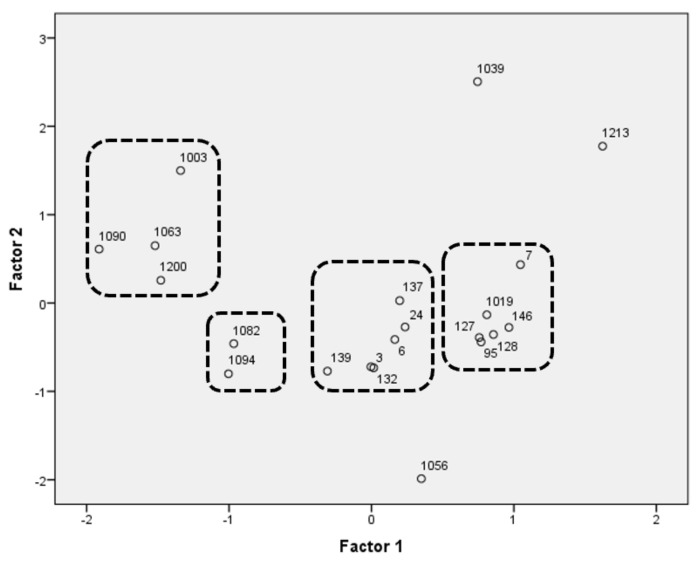
Principal component analysis run on the fitting parameters of the functional characteristics. Case projection (yeast distribution as a function of the variables). Dotted line represents groups of strains with the similar profile.

Table 5 shows the explained variance and the factor loadings, which describe the degree of correlation of the principal components with the functional traits examined. The analysis accounted for 77.61% of the total variability. The disposition of strains is represented in Figure 6, where there are four groups (two groups outline the behavior of the *Saccharomyces*, and the other two non-*Saccharomyces*) and three outliers. As a result of the PCA, five strains were selected as being representative of the whole population.

Strain 3, from the group in the center of the graph, was chosen thanks to its antioxidant properties, its enzymatic activities, and the high percentage of adhesion presented. Furthermore, in previous studies, this strain was used as a positive control thanks to its resistance to gastrointestinal conditions. Strain 146, from the group that includes the rest of the *Saccharomyces*, and strain 1039, an outlier, were chosen because of their antimycotic resistance and their relationship with the prebiotics studied (xylan or β-glucan). Moreover, strain 1039 also presented the best adhesion results. On the other hand, strains 1056 and 1094 were selected for their roles in the use of raffinose and trehalose. Strain 1056 also presented the lowest losses of viability and vitality after the attenuation process.

## 4. Conclusions

An adequate probiotic strain for human consumption must be chosen, not only for its probiotic properties, but also for other biotechnological features, depending on the purpose of the final product. This work investigated the functional and biotechnological capacities of twenty yeasts with probiotic character, and observed their behavior after an attenuation process that would allow the yeast to be safely incorporated into a food or supplement.

The strains with the best results in each test have been highlighted, so that the most appropriate strains can be chosen for specific uses. Five best strains were chosen based on PCA, taking into account the balance between all of the tests carried out. The strains are two strains of *H. osmophila* (1056 and 1094), one of *L. thermotolerans* (1039), and two of *S. cerevisiae* (3 and 146). Their results demonstrate their potential as health-promoting probiotics.

## Figures and Tables

**Table 1:** Yeast strains and their sources of isolation.

Species	Strains	Source
*Saccharomyces boulardii*	24	CP
*Saccharomyces cerevisiae*	3, 6, 7, 95, 127, 128, 132, 137, 139, 146	W
*Pichia kudriavzevii*	1003, 1200	W
*Pichia membranifaciens*	1019	W
*Lachancea thermotolerans*	1039	W
*Hanseniaspora osmophila*	1056, 1094	D
*Candida vini*	1063	W
*Pichia anomala*	1082, 1090	D
*Zygosaccharomyces bailii*	1213	FV

CP: commercial probiotic; W: winery; D: distillery; FV: fermented vegetables.

**Table 2:** Prebiotic metabolisms under aerobic and anaerobic conditions. Increase in absorbance at 600 nm between 0 and >72 h.

	STRAINS	Prebiotic/Assimilation Time								
		Aerobic Conditions								
		Cellulose	Inulin	Melibiose	Raffinose	Xylan	Trehalose	Pectin	Cellobiose	Beta-Glucans
*Saccharomyces*	3	-	-	-	0.56 (0–24]	-	-	0.50 (48–2]	-	-
	24	-	-	-	0.83 (0–24]	-	-	0.53 (48–72]	0.51 (48–72]	-
	6	-	-	-	0.93 (0–24]	-	0.53 > 72	0.55 (24–48]	0.50 (24–48]	0.54 (24–48]
	7	-	-	-	0.81 (0–24]	-	0.51 > 72	0.58 > 72	-	-
	95	-	-	-	0.84 (0–24]	-	-	0.53 > 72	-	-
	127	-	-	0.61 (24–48]	0.92 (0–24]	0.52 (48–72]	0.72 > 72	0.52 (48–72]	0.51 (48–72]	-
	128	-	-	0.52 (24–48]	0.99 (0–24]	-	0.58 > 72	0.53 (24–48]	0.61 (48–72]	-
	132	-	-	-	0.59 (0–24]	-	-	0.55 (48-–72]	-	-
	137	-	0.52 > 72	0.56 > 72	0.73 (0–24]	-	0.55 > 72	0.52 > 72	-	-
	139	-	0.61 (48–72]	0.52 > 72	0.97 (0–24]	-	0.67 (0–24]	0,.52 (48–72]	0.70 > 72	-
	146	-	-	0.50 > 72	1.16 (0–24]	-	-	0.50 > 72	0.60 > 72	-
Non-*Saccharomyces*	1003	-	-	0.69 (0–24]	0.72 (0–24]	0.58 (0–24]	0.74 (0–24]	0.89 (0–24]	0.59 (0–24]	1.14 (0–24]
	1019	-	-	-	0.69 (0–24]	-	-	-	-	-
	1039	-	-	0.75 (24–48]	0.86 (0–24]	0.72 (24–48]	0.70 (24–48]	0.74 > 72	0.54 (48–72]	0.58 (48–72]
	1056	-	-	-	0.56 (0–24]	-	-	-	-	0.76 (0–24]
	1063	-	-	-	0.53 (0–24]	0.56 (0–24]	0.54 (0–24]	0.55 (0–24]	0.86 (0–24]	0.95 (0–24]
	1082	-	0.56 (0–24]	-	0.53 (0–24]	0.55 (0–24]	-	0.80 (0–24]	0.89 (0–24]	0.89 (0–24]
	1090	-	0.88 (0–24]	0.66 (0–24]	-	0.55 (0–24]	0.63 (0–24]	0.96 (0–24]	1.08 (0–24]	1.12 (0–24]
	1094	0.6 (0–24]	0.73 (0–24]	0.94 (0–24]	0.86 (0–24]	0.77 (0–24]	1.56 (0–24]	0.94 (0–24]	1.60 (0–24]	1.27 (0–24]
	1200	-	0.61 > 72	-	-	-	0.50 (0–24]	0.82 (0–24]	0,.2 (0–24]	1.06 (0–24]
	1213	-	-	-	-	-	-	-	-	0.52 (24–48]
		**Anaerobic Conditions**								
		**Cellulose**	**Melibiose**	**Raffinose**	**Xylan**	**Trehalose**	**Cellobiose**	**Beta-Glucans**		
*Saccharomyces*	3	-	-	-	-	-	-	-		
	24	-	-	-	-	0.56 > 72	0.57 (24–48]	-		
	6	-	-	-	-	-	-	-		
	7	-	-	-	-	0.50 > 72	-	-		
	95	-	-	-	-	0.53 (48–72]	-	-		
	127	-	-	-	-	0.58 > 72	0.57 (48–72]	-		
	128	-	-	-	-	0.72 > 72	0.58 > 72	-		
	132	-	-	0.53 > 72	-	0.55 (48–72]	-	-		
	137	-	-	-	-	0.56 (48–72]	-	-		
	139	-	-	0.56 > 72	-	0.56 (48–72]	0.55 (0–24]	-		
	146	-	-	-	-	0.70 > 72	0.58 (0–24]	-		
Non-*Saccharomyces*	1003	-	-	-	-	-	0.50 (24–48]	-		
	1019	-	-	-	0.72 (24–48]	-	-	-		
	1039	0.50 (0–24]	-	-	-	-	0.51 (24–48]	0.68 (24–48]		
	1056	0.57 (24–48]	-	0.91 (0–24]	0.76 (48–72]	0.74 (24–48]	-	-		
	1063	-	-	-	0.64 (24–48]	-	0.54 (24–48]	0.64 (24–48]		
	1082	-	-	0.51 (24–48]	0.56 (0–24]	-	-	-		
	1090	-	-	-	0.58 (48–72]	-	-	-		
	1094	-	-	-	-	-	0.58 (0–24]	-		
	1200	-	-	-	-	0.54 > 72	-	-		
	1213	-	0,65 (48–72]	-	0.50 (24–48]	-	-	-		

Prebiotics that were not used by any strain have been omitted. “-” Means prebiotic was not used.

**Table 3:** Qualitative analysis of the enzymatic activity shown by the yeasts.

	Strain	Enzyme Studied *										
		02	03	04	05	06	07	08	09	10	11	12
*Saccharomyces cerevisiae*	3	-	+	+	-	+	+	+	-	-	+	+
	6	-	-	-	-	+	-	-	-	-	-	+
	7	-	+	+	-	+	-	-	-	-	+	+
	24	-	+	+	-	+	-	-	-	-	+	+
	95	-	+	+	-	+	-	-	-	-	-	+
	127	-	+	+	-	+	-	-	-	-	+	+
	128	-	+	+	-	+	-	-	-	-	+	+
	132	-	-	-	-	+	-	-	-	-	+	+
	137	-	+	-	-	+	-	-	-	-	+	+
	139	-	+	+	-	+	-	-	-	-	+	+
	146	-	-	-	-	+	-	-	-	-	+	+
Non-*Saccharomyces*	1003	-	+	+	-	+	-	-	-	-	+	+
	1019	+	+	+	-	+	-	-	-	-	+	+
	1039	-	-	-	-	+	-	-	-	-	-	+
	1056	-	-	-	-	+	-	-	-	-	+	+
	1063	-	+	+	+	+	-	-	-	-	+	+
	1082	-	+	-	-	+	-	-	-	-	+	+
	1090	-	+	+	-	+	-	-	-	-	+	+
	1094	-	+	+	-	+	-	-	-	-	+	+
	1200	-	+	+	-	+	-	-	-	-	-	+

* 02: Alkaline phosphatase; 03: esterase (C 4); 04: esterase lipase (C 14); 05: lipase; 06: leucine arylamidase; 07: valine arylamidase; 08: cystine arylamidase; 09: trypsin; 10: α-chymotrypsin; 11: acid phosphatase; 12: naphthol-AS-BI-phosphohydrolase. Positive activity: “+”; activity not detected: “-”.

**Table 4:** Viability loss percentage for different proven conditions in the attenuation setup.

Amplitude (%)	Total Time (min)	Pulses (s)		Initial Population 10^7^ cfu/mL	Initial Population 10^6^ cfu/mL
		On	Off		
80	3	-	-	58.5%	60.9%
80	2	2	10	47.1%	45.4%
80	1	2	10	3.1%	2.1%
60	3	-	-	37.7%	42.6%
60	3	2	10	26.9%	30.2%
60	2	2	10	6.9%	6.1%
60	1	2	10	0%	2.2%

**Table 5:** Contributions of the highly correlated variables and their loadings in principal components 1, 2, 3, 4, and 5.

Principal Component	Variance Explained (%)	Total Variance (%)	Most Highly Correlated	Loading
1	27.38	27.38	Fluconazol PI Canesten PI Mycostatin TI Ciclochem PI Pectin	0.87 0.80 0.71 −0.70 −0.55
2	20.38	47.77	Fluconazol TI Raffinose Canesten TI Trehalose	0.81 −0.80 0.79 −0.69
3	11.34	59.11	β-glucan Xylan Cellulose DPPH Attenuation vital.	0.79 0.78 0.77 −0.73 −0.56
4	10.04	69.15	Ciclochem TI Cellobiose Melibiose	0.85 0.72 −0.55
5	8.46	77.61	Cholesterol Attenuation	0.88 −0.62

PI: partian inhibition; TI: total inhibition.

## Data Availability

Not applicable.
